# An Intelligent Parkinson's Disease Diagnostic System Based on a Chaotic Bacterial Foraging Optimization Enhanced Fuzzy KNN Approach

**DOI:** 10.1155/2018/2396952

**Published:** 2018-06-21

**Authors:** Zhennao Cai, Jianhua Gu, Caiyun Wen, Dong Zhao, Chunyu Huang, Hui Huang, Changfei Tong, Jun Li, Huiling Chen

**Affiliations:** ^1^School of Computer Science and Engineering, Northwestern Polytechnical University, Xi'an 710072, China; ^2^Department of Radiology, The First Affiliated Hospital of Wenzhou Medical University, Wenzhou, Zhejiang 325035, China; ^3^College of Computer Science and Technology, Changchun Normal University, Changchun 130032, China; ^4^College of Computer Science and Technology, Changchun University of Science Technology, Changchun 130032, China; ^5^College of Mathematics, Physics and Electronic Information Engineering, Wenzhou University, Wenzhou, Zhejiang 325035, China

## Abstract

Parkinson's disease (PD) is a common neurodegenerative disease, which has attracted more and more attention. Many artificial intelligence methods have been used for the diagnosis of PD. In this study, an enhanced fuzzy* k*-nearest neighbor (FKNN) method for the early detection of PD based upon vocal measurements was developed. The proposed method, an evolutionary instance-based learning approach termed CBFO-FKNN, was developed by coupling the chaotic bacterial foraging optimization with Gauss mutation (CBFO) approach with FKNN. The integration of the CBFO technique efficiently resolved the parameter tuning issues of the FKNN. The effectiveness of the proposed CBFO-FKNN was rigorously compared to those of the PD datasets in terms of classification accuracy, sensitivity, specificity, and AUC (area under the receiver operating characteristic curve). The simulation results indicated the proposed approach outperformed the other five FKNN models based on BFO, particle swarm optimization, Genetic algorithms, fruit fly optimization, and firefly algorithm, as well as three advanced machine learning methods including support vector machine (SVM), SVM with local learning-based feature selection, and kernel extreme learning machine in a 10-fold cross-validation scheme. The method presented in this paper has a very good prospect, which will bring great convenience to the clinicians to make a better decision in the clinical diagnosis.

## 1. Introduction

Parkinson's disease (PD), a degenerative disorder of the central nervous system, is the second most common neurodegenerative disease [1]. The number of people suffering from PD has increased rapidly worldwide [2], especially in developing countries in Asia [3]. Although its underlying cause is unknown, the symptoms associated with PD can be significantly alleviated if detected in the early stages of illness [4–6]. PD is characterized by tremors, rigidity, slowed movement, motor symptom asymmetry, and impaired posture [7, 8]. Research has shown phonation and speech disorders are also common among PD patients [9]. In fact, phonation and speech disorders can appear in PD patients as many as five years before being clinically diagnosed with the illness [10]. The voice disorders associated with PD include dysphonia, impairment in vocal fold vibration, and dysarthria, disability in correctly articulating speech phonemes [11, 12]. Little et al. [13] first attempted to identify PD patients with dysphonic indicators using a combination of support vector machines (SVM), efficient learning machines, and the feature selection approach. The study results indicated that the proposed method efficiently identified PD patients with only four dysphonic features.

Inspired by the results obtained by Little et al. [13], many other researchers conducted studies on the use of machine learning techniques to diagnose PD patients on the same dataset (hereafter Oxford dataset). In [14], Das made a comparison of classification score for diagnosis of PD between artificial neural networks (ANN), DMneural, and Regression and Decision Trees. The ANN classifier yielded the best results of 92.9%. In [15], AStröm et al. designed a parallel feed-forward neural network system and yielded an improvement of 8.4% on PD classification. In [16], Sakar et al. proposed a method that combined SVM and feature selection using mutual information to detect PD and obtained a classification accuracy of 92.75%. In [17], a PD detection method developed by Li et al. using an SVM and a fuzzy-based nonlinear transformation method yielded a maximum classification accuracy of 93.47%. In another study, Shahbaba et al. [18] compared the classification accuracies of a nonlinear model based on a combination of the Dirichlet processes, multinomial logit models, decision trees, and support vector machines, which yielded the highest classification score of 87.7%. In [19], Psorakis et al. put forward novel convergence methods and model improvements for multiclass mRVMs. The improved model achieved an accuracy of 89.47%. In [20], Guo et al. proposed a PD detection method with a maximum classification accuracy of 93.1% by combination of genetic programming and the expectation maximization algorithm (GP-EM). In [21], Luukka used a similarity classifier and a feature selection method using fuzzy entropy measures to detect PD, and a mean classification accuracy of 85.03% is achieved. In [22], Ozcift et al. presented rotation forest ensemble classifiers with feature selection using the correlation method to identify PD patients; the proposed model yielded a highest classification accuracy of 87.13%. In [23], Spadoto et al. used a combination of evolutionary-based techniques and the Optimum-Path Forest (OPF) classifier to detect PD with a maximum classification accuracy of 84.01%. In [24], Polat integrated fuzzy C-means clustering-based feature weighting (FCMFW) into a KNN classifier, which yielded a PD classification accuracy of 97.93%. In [25], Chen et al. combined a fuzzy* k*-nearest neighbor classifier (FKNN) with the principle component analysis (PCA-FKNN) method to detect PD; the proposed diagnostic system yielded a maximum classification accuracy of 96.07%. In [26], Zuo et al. developed an PSO-enhanced FKNN based PD diagnostic system with a mean classification accuracy of 97.47%. In [27–29], Babu et al. proposed a ‘projection based learning meta-cognitive radial basis function network (PBL-McRBFN)' approach for the prediction of PD, which obtained an testing accuracy of 96.87% on the gene expression data sets, 99.35% on standard vocal data sets, 84.36% on gait PD data sets, and 82.32% on magnetic resonance images. In [30], the hybrid intelligent system for PD detection was proposed which included several feature preprocessing methods and classification techniques using three supervised classifiers such as least-square SVM, probabilistic neural networks, and general regression neural network; the experimental results gives a maximum classification accuracy of 100% for the PD detection. Furthermore, in [31], Gök et al. developed a rotation forest ensemble KNN classifier with a classification accuracy of 98.46%. In [32], Shen et al. proposed an enhanced SVM based on fruit fly optimization algorithm, and have achieved 96.90% classification accuracy for diagnosis of PD. In [33], Peker designed a minimum redundancy maximum relevance (mRMR) feature selection algorithm with the complex-valued artificial neural network to diagnosis of PD, and obtained a classification accuracy of 98.12%. In [34], Chen et al. proposed an efficient hybrid kernel extreme learning machine with feature selection approach. The experimental results showed that the proposed method can achieve the highest classification accuracy of 96.47% and mean accuracy of 95.97% over 10 runs of 10-fold CV. In [35], Cai et al. have proposed an optimal support vector machine (SVM) based on bacterial foraging optimization (BFO) combined with the relief feature selection to predict PD, the experimental results have demonstrated that the proposed framework exhibited excellent classification performance with a superior classification accuracy of 97.42%.

Different from the work of Little et al., Sakar et al. [36] designed voice experiments with sustained vowels, words, and sentences from PD patients and controls. The paper reported that sustained vowels had more PD-discriminative power than the isolated words and short sentences. The study result achieved 77.5% accuracy by using SVM classifier. From then on, several works have been proposed to detect PD using this PD dataset (hereafter Istanbul dataset). Zhang et al. [37] proposed a PD classification algorithm that integrated a multi-edit-nearest-neighbor algorithm with an ensemble learning algorithm. The algorithm achieved higher classification accuracy and stability compared with the other algorithms. Abrol et al. [38] proposed a kernel sparse greedy dictionary algorithm for classification tasks, comparing with kernel K-singular value decomposition algorithm and kernel multilevel dictionary learning algorithm. The method achieved an average classification accuracy of 98.2% and the best accuracy of 99.4% on the Istanbul PD dataset with multiple types of sound recordings. In [39], the authors investigated six classification algorithms, including Adaboost, support vector machines, neural network with multilayer perceptron (MLP) structure, ensemble classifier, K-nearest neighbor, naive Bayes, and presented feature selection algorithms including LASSO, minimal redundancy maximal relevance, relief, and local learning-based feature selection on the Istanbul PD dataset. The paper indicated that applying feature selection methods greatly increased the accuracy of classification. The SVM and KNN classifiers with local learning-based feature selection obtained the optimum prediction ability and execution times.

As shown above, ANN and SVM have been extensively applied to the detection of PD. However, understanding the underlying decision-making processes of ANN and SVM is difficult due to their black-box characteristics. Compared to ANN and SVM, FKNN is much simpler and yield more easily interpretable results. FKNN [40, 41] classifiers, improved versions of traditional* k*-nearest neighbor (KNN) classifiers, have been studied extensively since first proposed for the use of diagnostic purposes. In recent years, many variant versions of KNNs based on fuzzy sets theory and several extensions have been developed, such as fuzzy rough sets, intuitionistic fuzzy sets, type 2 fuzzy sets, and possibilistic theory based KNN [42]. FKNN allows for the representation of imprecise knowledge via the introduction of fuzzy measures, providing a powerful method of similarity description among instances. In FKNN methods, fuzzy set theories are introduced into KNNs, which assign membership degrees to different classes instead of the distances to their* k*-nearest neighbors. Thus, each of the instances is assigned a class membership value rather than binary values. When it comes to the voting stage, the highest class membership function value is selected. Then based on these properties, FKNN has been applied to numerous practical problems, such as medical diagnosis problems [25, 43], protein identification and prediction problems [44, 45], bankruptcy prediction problems [46], slope collapse prediction problems [47], and grouting activity prediction problems [48].

The classification performance of an FKNN greatly relies on its tuning parameters, neighborhood size (*k*), and fuzzy strength (*m*). Therefore, the two parameters should be precisely determined before applying FKNN to practical problems. Several studies concerning parameter tuning in FKNN have been conducted. In [46], Chen et al. presented the particle swarm optimization (PSO) based method to automatically search for the two tuning parameters of an FKNN. According to the results of the study, the proposed method could be effectively and efficiently applied to bankruptcy prediction problems. More recently, Cheng et al. [48] developed a differential evolution optimization approach to determine the most appropriate tuning parameters of an FKNN and successfully applied to grouting activity prediction problems in the construction industry. Later, Cheng et al. [47] proposed using firefly algorithm to tune the hyperparameters of the FKNN model. The FKNN model was then applied to slop collapse prediction problems. The experiment results indicated that the developed method outperformed other common algorithms. The bacterial foraging optimization (BFO) method [49], a relatively new swarm-intelligence algorithm, mimics the cooperative foraging behavior of several bacteria on a multidimensional continuous search space and, therefore, effectively balances exploration and exploitation events. Since its introduction, BFO has been subtly introduced to real-world optimization problems [50–55], such as optimal controller design problems [49], stock market index prediction problems [56], automatic circle detection problems involving digital images [57], harmonic estimation problems [58], active power filter design problems [59], and especially the parameter optimization of machine learning methods [60–63]. In [60], BFO was introduced to wavelet neural network training and applied successfully to load forecasting. In [61], an improved BFO algorithm was proposed to fine-tune the parameters of fuzzy support vector machines to identify the fatigue status of the electromyography signal. The experimental results have shown that the proposed method is an effective tool for diagnosis of fatigue status. In [62], BFO was proposed to learn the structure of Bayesian networks. The experimental results verify that the proposed BFO algorithm is a viable alternative to learn the structures of Bayesian networks and is also highly competitive compared to state-of-the-art algorithms. In [63], BFO was employed to optimize the training parameters appeared in adaptive neuro-fuzzy inference system for speed control of matrix converter- (MC-) fed brushless direct current (BLDC) motor. The simulation results have reported that the BFO approach is much superior to the other nature-inspired algorithms. In [64], a chaotic local search based BFO (CLS-BFO) was proposed, which introduced the DE operator and the chaotic search operator into the chemotaxis step of the original BFO.

Inspired from the above works, in this paper, the BFO method was integrated with FKNN for the maximum classification performance. In order to further improve the diversity of the bacteria swarm, chaos theory combination with the Gaussian mutation was introduced in BFO. Then, the resulting CBFO-FKNN model was applied to the detection of PD. In our previous work, we have applied BFO in the classification of speech signals for PD diagnosis [35]. In this work, we have further improved the BFO by embedding the chaotic theory and Gauss mutation and combined with the effective FKNN classifier. In order to validate the effectiveness of the proposed CBFO-FKNN approach, FKNN based on five other meta-heuristic algorithms including original BFO, particle swarm optimization (PSO), genetic algorithms (GA), fruit fly optimization (FOA), and firefly algorithm (FA) was implemented for strict comparison. In addition, advanced machine learning methods, including the support vector machine (SVM), kernel based extreme learning machine (KELM) methods, and SVM with local learning-based feature selection (LOGO) [65] (LOGO-SVM), were compared with the proposed CBFO-FKNN model in terms of classification accuracy (ACC), area under the receiver operating characteristic curve (AUC), sensitivity, and specificity. The experimental results show that the proposed CBFO-FKNN approach has exhibited high ACC, AUC, sensitivity, and specificity on both datasets. This work is a fully extended version of our previously published conference paper [66] and that further improved method has been provided.

The main contributions of this study are as follows:

- First, we introduce chaos theory and Gaussian mutation enhanced BFO to adaptively determine the two key parameters of FKNN, which aided the FKNN classifier in more efficiently achieving the maximum classification performance, more stable and robust when compared to five other bio-inspired algorithms-based FKNN models and other advanced machine learning methods such as SVM and KELM.
- The resulting model, CBFO-FKNN, is introduced to discriminate the persons with PD from the healthy ones on the two PD datasets of UCI machine learning repository. It is promising to serve as a computer-aided decision-making tool for early detection of PD.

The remainder of this paper is structured as follows. In Section 2, background information regarding FKNN, BFO, chaos theory, and Gaussian mutation is presented. The implementation of the proposed methodology is explained in Section 3. In Section 4, the experimental design is described in detail. The experimental results and a discussion are presented in Section 5. Finally, Section 6 concludes the paper.

## 2. Background Information

### 2.1. Fuzzy* k*-Nearest Neighbor (FKNN)

In this section, a brief description of FKNN is provided. A detailed description of FKNN can be referred to in [41]. In FKNN, the fuzzy membership values of samples are assigned to different categories as follows:

(1) $$\begin{array}{c} u_{i}\left(\;x\;\right)=\frac{\sum_{j=1}^{K}u_{ij}\left(1/{\left\|x-x_{j}\right\|}^{2/\left(m-1\right)}\right)}{\sum_{j=1}^{K}\left(1/{\left\|x-x_{j}\right\|}^{2/\left(m-1\right)}\right)} \end{array}$$

where* i*=1,2,…*C*,* j*=1,2,…,*K*,* C* represents the number of classes, and* K* means the number of nearest neighbors. The fuzzy strength parameter (*m*) is used to determine how heavily the distance is weighted when calculating each neighbor's contribution to the membership value. *m* ∈ (1, *∞*). ‖*x* − *x*_*j*_‖ is usually selected as the value of* m*. In addition, the Euclidean distance, the distance between* x* and its* j*th nearest neighbor *x*_*j*_, is usually selected as the distance metric. Furthermore, *u*_*ij*_ denotes the degree of membership of the pattern *x*_*j*_ from the training set to class* i* among the* k*-nearest neighbors of *x*. In this study, the constrained fuzzy membership approach was adopted in that the* k*-nearest neighbors of each training pattern (i.e., *x*_*k*_) were determined, and the membership of* x*_*k*_ in each class was assigned as

(2) $$\begin{array}{c} u_{ij}\left(\;x_{k}\;\right)=\left\{\begin{array}{cc} 0.51+\left(\frac{n_{j}}{K}\right)*0.49, & \text{if }j=i\\ \left(\frac{n_{j}}{K}\right)*0.49, & \text{if }j\neq i. \end{array}\right. \end{array}$$

The value of *n*_*j*_ denotes the number of neighbors belonging to *j*^th^ class. The membership values calculated using (2) should satisfy the following equations:

(3) ∑I=1Cμij=1,j=1,2,⋯,n,  C  is  the  number  of  classes0<∑j=1nuij<n,uij∈0,1.

After calculating all of the membership values of a query sample, it is assigned to the class with which it has the highest degree of membership, i.e.,

(4) $$\begin{array}{c} C\left(\;x\;\right)=\overset{C}{\underset{i=1}{arg\;max}}\left(\;u_{i}\left(\;x\;\right)\;\right) \end{array}$$

### 2.2. Bacterial Foraging Optimization (BFO)

The bacterial foraging algorithm (BFO) is a novel nature-inspired optimization algorithm proposed by Passino in 2002 [49]. The BFO simulates the mechanism of approaching or moving away while sensing the concentration of peripheral substances in bacterial foraging process. This method contains four basic behaviors: chemotaxis, swarming, reproduction, and elimination-dispersal.

#### 2.2.1. Chemotaxis

The chemotaxis behavior simulates two different positional shifts of* E. coli* bacterium that depend on the rotation of the flagellum, namely, tumbling and moving. The tumbling refers to looking for new directions and the moving refers to keeping the direction going. The specific operation is as follows: first, a unit step is moved in a certain random direction. If the fitness value of the new position is more suitable than the previous one, it will continue to move in that direction; if the fitness value of the new position is not better than before, the tumble operation is performed and moves in another random direction. When the maximum number of attempts is reached, the chemotaxis step is stopped. The chemotaxis step to operate is indicated by the following:

(5) θij+1,k,l=θij,k,l+Ci∗dctidcti=ΔiΔTiΔi

where *θ*^*i*^(*j*, *k*, *l*) is the position of the* i*th bacterium. The* j*,* k*, and* l*, respectively, indicate the number of bacterial individuals to complete the chemotaxis, reproduction, and elimination-dispersal.* C*(*i*) is the chemotaxis step length for the* i*th bacteria to move. Δ is the random vector between [-1, 1].

#### 2.2.2. Swarming

In the process of foraging, the bacterial community can adjust the gravitation and repulsion between the cell and the cell, so that the bacteria in the case of aggregation characteristics and maintain their relatively independent position. The gravitation causes the bacteria to clump together, and the repulsion forces the bacteria to disperse in a relatively independent position to obtain food.

#### 2.2.3. Reproduction

In the reproduction operation of BFO algorithm, the algorithm accumulates the fitness values of all the positions that the bacterial individual passes through in the chemotaxis operation and arranges the bacteria in descending order. Then the first half of the bacteria divides themselves into two bacteria by binary fission, and the other half die. As a result, the new reproduced bacterial individual has the same foraging ability as the original individual, and the population size of bacterial is always constant.

#### 2.2.4. Elimination-Dispersal

After the algorithm has been reproduced for several generations, the bacteria will undergo elimination-dispersal at a given probability* Ped*, and the selected bacteria will be randomly redistributed to new positions. Specifically, if a bacterial individual in the bacterial community satisfies the probability* Ped *of elimination-dispersal, the individual loses the original position of foraging and randomly selects a new position in the solution space, thereby promoting the search of the global optimal solution.

### 2.3. Chaotic Mapping

Chaos, as a widespread nonlinear phenomenon in nature, has the characteristics of randomness, ergodicity, sensitivity to initial conditions and so on [67]. Due to the characteristics of ergodicity and randomness, chaotic motions can traverse all the states in a certain range according to their own laws without repetition. Therefore, if we use chaos variables to search optimally, we will undoubtedly have more advantages than random search. Chaos ergodicity features can be used to optimize the search and avoid falling into the local minima; therefore, chaos optimization search method has become a novel optimization technique. Chaotic sequences generated by different mappings can be used such as logistic map, sine map, singer map, sinusoidal map, and tent map. In this paper, several chaotic maps were tried and the best one was chosen to combine with the BFO algorithm. According to the preliminary experiment, logistic map has achieved the best results. Thus, the chaotic sequences are generated by using logistic map as follows:

(6) $$\begin{array}{c} x_{i+1}=ux_{i}\left(\;1-x_{i}\;\right) \end{array}$$

*u* is the control parameter and let* u* = 4. When* u* = 4, the logistic mapping comes into a thorough chaotic state. Let *x*_*i*_ ∈ (0,1) and *x*_*i*_ ≠ 0.25,0.5,0.75.

The initial bacterial population *θ* is mapped to the chaotic sequence that has been generated according to (6), resulting in a corresponding chaotic bacterial population* pch*.

(7) $$\begin{array}{c} pch=x_{i}*\theta \end{array}$$

### 2.4. Gaussian Mutation

The Gaussian mutation operation has been derived from the Gaussian normal distribution and has demonstrated its effectiveness with application to evolutionary search [68]. This theory was referred to as classical evolutionary programming (CEP).The Gaussian mutations have been used to exploit the searching capabilities of ABC [69], PSO [70], and DE [71]. Also, Gaussian mutation is more likely to create a new offspring near the original parent because of its narrow tail. Due to this, the search equation will take smaller steps allowing for every corner of the search space to be explored in a much better way. Hence it is expected to provide relatively faster convergence. The Gaussian density function is given by

(8) $$\begin{array}{c} f_{gaussian\left(0,\sigma^{2}\right)}\left(\;\alpha \;\right)=\frac{1}{\sqrt{2\pi \sigma^{2}}}e^{-\alpha^{2}/2\sigma^{2}} \end{array}$$

where *σ*^2^ is the variance for each member of the population.

## 3. Proposed CBFO-FKNN Model

**Figure 1:**
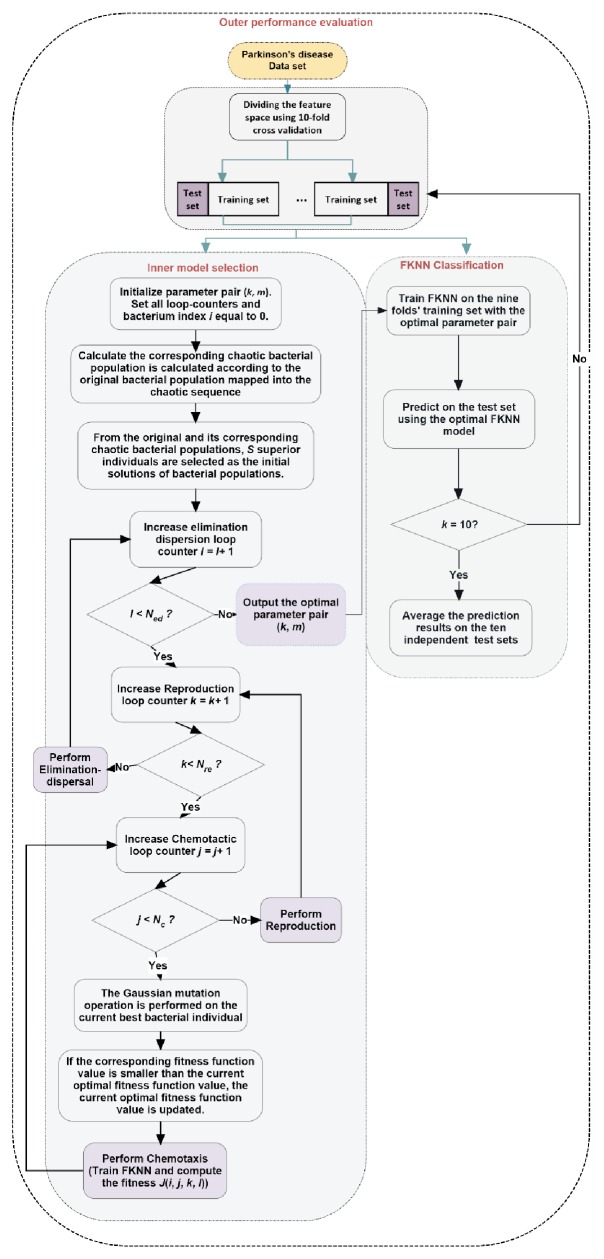
Flowchart of the proposed CBFO-FKNN diagnostic system.

In this section, we described the new evolutionary FKNN model based on the CBFO strategy. The two key parameters of FKNN were automatically tuned based on the CBFO strategy. As shown in Figure 1, the proposed methodology has two main parts, including the inner parameter optimization procedure and outer performance evaluation procedure. The main objective of the inner parameter optimization procedure was to optimize the parameter neighborhood size (*k*) and fuzzy strength parameter (*m*) by using the CBFO technique via a 5-fold cross-validation (CV). Then, the obtained best values of (*k*,* m*) were input into the FKNN prediction model in order to perform the PD diagnostic classification task in the outer loop via the 10-fold CV. The classification error rate was used as the fitness function.

(9) $$\begin{array}{c} fitness=\frac{\left(\sum_{i=1}^{K}testError_{i}\right)}{k} \end{array}$$

where* testError*_*i*_ means the average test error of the FKNN classifier.

The main steps conducted by the CBFO strategy are described in detail as shown in Algorithm 1.

## 4. Experimental Design

### 4.1. Oxford Parkinson's Disease Data

The Oxford Parkinson's disease data set was donated by Little et al. [13], abbreviation as Oxford dataset. The data set was used to discriminate patients with PD from healthy controls via the detection of differences in vowel sounds. Various biomedical voice measurements were collected from 31 subjects. 23 of them are patients with PD, and 8 of them are healthy controls. The subjects ranged from 46 to 85 years of age. Each subject provided an average of six sustained vowel “ahh…” phonations, ranging from 1 to 36 seconds in length [13], yielding 195 total samples. Each recording was subjected to different measurements, yielding 22 real-value features.  Table 1 lists these 22 vocal features and their statistical parameters.

### 4.2. Istanbul Parkinson's Disease Data

The second data set in this study was deposited by Sakar et al. [36] from Istanbul, Turkey, abbreviation as Istanbul dataset. It contained multiple types of sound recordings, including sustained vowels, numbers, words, and short sentences from 68 subjects. Specifically, the training data collected from 40 persons including 20 patients with PD ranging from 43 to 77 and 20 healthy persons ranging from 45 to 83, while testing data was collected from 28 different patients with PD ranging 39 and 79. In this study, we selected only 3 types of sustained vowel recordings /a/, /o/, and /u/, with similar data type to the Oxford PD dataset. We merged them together and produced a database which contains total 288 sustained vowels samples and the analyses were made on these samples. As shown in Table 2, a group of 26 linear and time-frequency based features are extracted for each voice sample.

### 4.3. Experimental Setup

The experiment was performed on a platform of Windows 7 operating system with an Intel (R) Xeon (R) CPU E5-2660 v3 @ 2.6 GHz and 16GB of RAM. The CBFO-FKNN, BFO-FKNN, PSO-FKNN, GA-FKNN, FOA-FKNN, FA-FKNN, SVM, and KELM classification models were implemented with MATLAB 2014b. The LIBSVM package [72] was used for the SVM classification. The algorithm available at http://www3.ntu.edu.sg/home/egbhuang was used for the KELM classification. The CBFO-FKNN method was implemented from scratch. The data was scaled into a range of [0, 1] before each classification was conducted.

The parameters* C* and *γ* in *K*(*x*, *x*_*i*_) = exp(−*γ*‖*x* − *x*_*i*_‖^2^) used during the SVM and KELM classifications were determined via the grid search method; the search ranges were defined as *C* ∈ {2^−5^, 2^−3^,…, 2^15^} and *γ* ∈ {2^−15^, 2^−13^,…, 2^5^}. A population swarm size of 8, chemotactic step number of 25, swimming length of 4, reproduction step number of 3, elimination-dispersal event number of 2, and elimination-dispersal probability of 0.25 were selected for the CBFO-FKNN. The chemotaxis step value was established through trial and error, as shown in the experimental results section. The initial parameters of the other four meta-heuristic algorithms involved in training FKNN are chosen by trial and error as reported in Table 3.

### 4.4. Data Classification

A stratified* k*-fold CV [73] was used to validate the performance of the proposed approach and other comparative models. In most studies,* k* is given the value of 10. During each step, 90% of the samples are used to form a training set, and the remaining samples are used as the test set. Then, the average of the results of all 10 trials is computed. The advantage of this method is that all of the test sets remain independent, ensuring reliable results.

A nested stratified 10-fold CV, which has been widely used in previous research, was used for the purposes of this study [74]. The classification performance evaluation was conducted in the outer loop. Since a 10-fold CV was used in the outer loop, the classifiers were evaluated in one independent fold of data, and the other nine folds of data were left for training. The parameter optimization process was performed in the inner loop. Since a 5-fold CV was used in the inner loop, the CBFO-FKNN searched for the optimal values of* k* and* m*, and the SVM and KELM searched for the optimal values of* C* and *γ* in the remaining nine folds of data. The nine folds of data were further split into one fold of data for the performance evaluation, and four folds of data were left for training.

### 4.5. Evaluation Criteria

ACC, AUC, sensitivity, and specificity were taken to evaluate the performance of different models. These measurements are defined as

(10) ACC=TP+TNTP+FP+FN+TN×100%

(11) Sensitivity=TPTP+FN×100%

(12) Specificity=TNFP+TN×100%

where TP is the number of true positives, FN means the number of false negatives, TN represents the true negatives, and FP is the false positives. AUC [75] is the area under the ROC curve.

## 5. Experimental Results and Discussion

### 5.1. Benchmark Function Validation

In order to test the performance of the proposed algorithm CBFO, 23 benchmark functions which include unimodal, multimodal, and fixed-dimension multimodal were used to do experiments. These functions are listed in Tables 4–6 where Dim represents the dimension, Range is the search space, and *f*_min_ is the best value.

In order to verify the validity of the proposed algorithm, the original BFO, Firefly Algorithm(FA)[76], Flower Pollination Algorithm (FPA)[77], Bat Algorithm (BA)[78], Dragonfly Algorithm (DA)[79], Particle Swarm Optimization (PSO)[80], and the improved BFO called PSOBFO were compared on these issues. The parameters of the above algorithm are set according to their original papers, and the specific parameter values are set as shown in Table 7. In order to ensure that the results obtained are not biased, 30 independent experiments are performed. In all experiments, the number of population size is set to 50 and the maximum number of iterations is set to 500.

**Figure 2:**
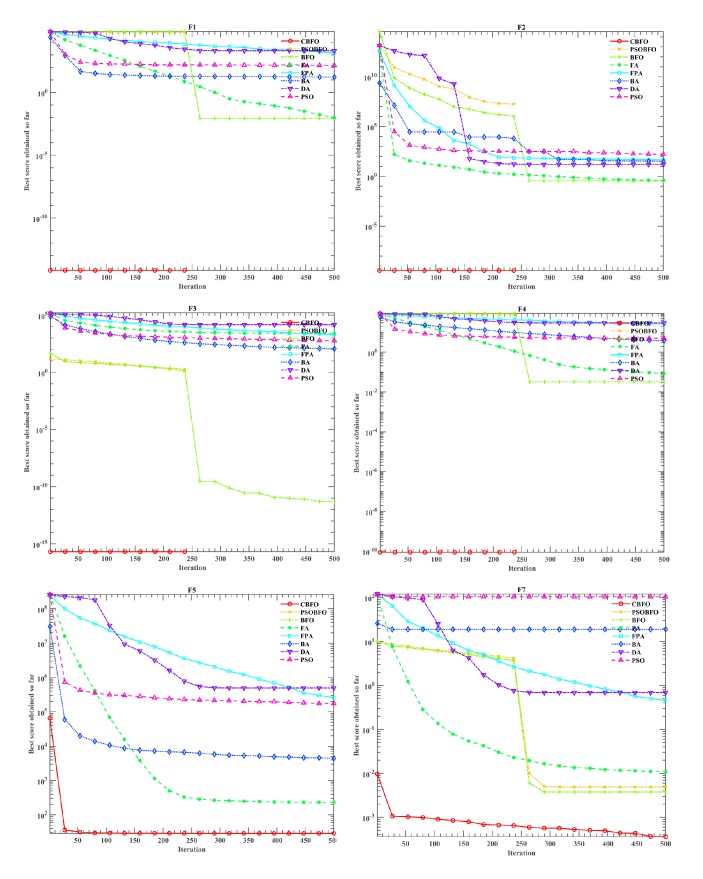
Convergence curves of unimodal functions.

**Figure 3:**
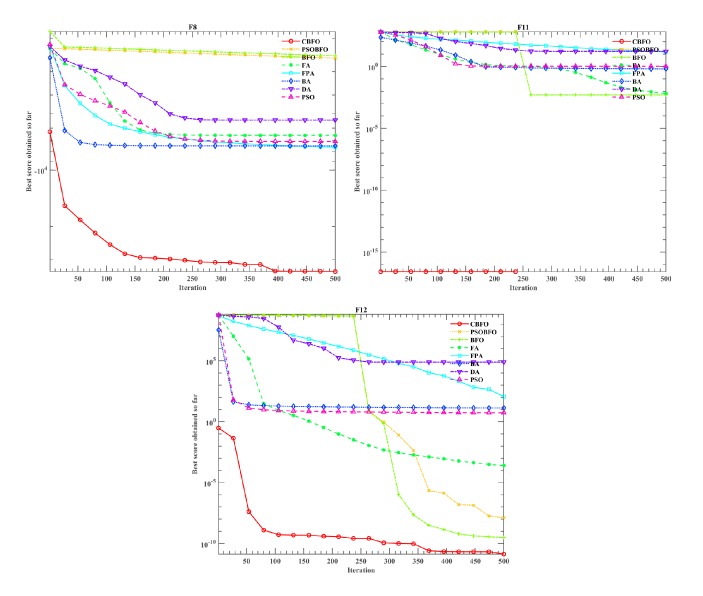
Convergence curves of multimodal functions.

**Figure 4:**
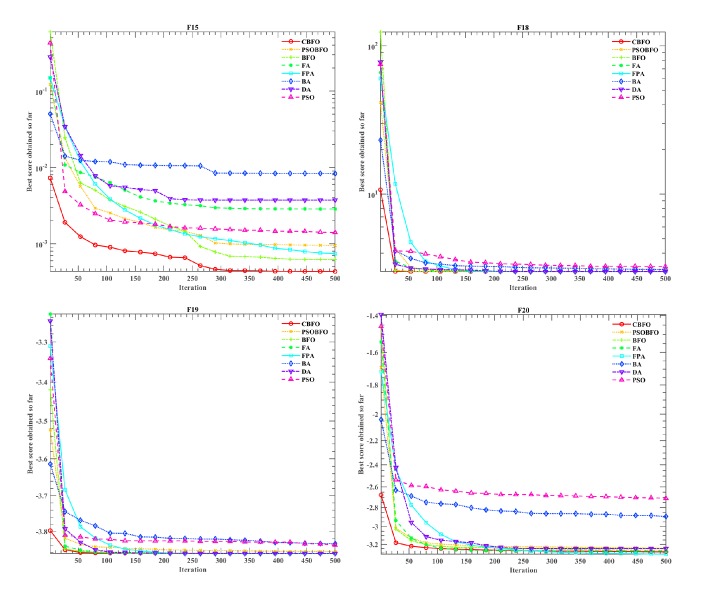
Convergence curves based on fixed-dimension multimodal functions.

Tables 8–10 show average results (Avg), standard deviation (Stdv), and overall ranks for different algorithms dealing with F1-23 issues. It should be noted that the ranking is based on the average result (Avg) of 30 independent experiments for each problem. In order to visually compare the convergence performance of our proposed algorithm and other algorithms, Figures 2–4 use the logarithmic scale diagram to reflect the convergence behaviors. In Figures 2–4, we only select typical function convergence curves from unimodal functions, multimodal functions, and fixed-dimension multimodal functions, respectively. The results of the unimodal F1-F7 are shown in Table 8. As shown, the optimization effect of CBFO in F1, F2, F3, and F4 is the same as the improved PSOBFO, but the performance is improved compared with the original BFO. Moreover, From the ranking results, it can be concluded that, compared with other algorithms, CBFO is the best solution to solve the problems of F1-F7.

With respect to the convergence trends described in Figure 2, it can be observed that the proposed CBFO is capable of testifying a very fast convergence and it can be superior to all other methods in dealing with F1, F2, F3, F4, F5, and F7. For F1, F2, F3, and F4, the CBFO has converged so fast during few searching steps compared to other algorithms. In particular, when dealing with cases F1, F2, F3, and F4, the trend converges rapidly after 250 iterations.

The calculated results for multimodal F8-F13 are tabulated in Table 9. It is observed that CBFO has attained the exact optimal solutions for 30-dimension problems F8 and F12 in all 30 runs. From the results for F9, F10, F11, and F13 problems, it can be agreed that the CBFO yields very competitive solutions compared to the PSOBFO. However, based on rankings, the CBFO is the best overall technique and the overall ranks show that the BFO, FA, BA, PSO, FPA, and DA algorithms are in the next places, respectively.

According to the corresponding convergence trend recorded in Figure 3, the relative superiority of the proposed CBFO in settling F8, F11, and F12 test problems can be recognized. In tackling F11, the CBFO can dominate all its competitors in tackling F11 only during few iterations. On the other hand, methods such as FPA, BA, DA, and PSO still cannot improve the quality of solutions in solving F11 throughout more steps.

The results for F14 to F23 are tabulated in Table 10. The results in Table 10 reveal that the CBFO is the best algorithm and can outperform all other methods in dealing with F15 problems. In F16, F17, and F19, it can be seen that the optimization effect of all the algorithms is not much different. In dealing with F20 case, the CBFO's performance is improved compared to original BFO and the improved PSOBFO. Especially in solving F18, the proposed algorithm is much better than the improved PSOBFO. From Figure 4, we can see that the convergence speed of the CBFO is better than other algorithms in dealing with F15, F18, F19, and F20. For F15, it surpasses all methods.

In order to investigate significant differences of obtained results for the CBFO over other competitors, the Wilcoxon rank-sum test [81] at 5% significance level was also employed in this paper. The p values of comparisons are reported in Tables 11–13. In each table, each p value which is not lower than 0.05 is shown in bold face. It shows that the differences are not significant.

The p values are also provided in Table 11 for F1-F7. Referring to the p values of the Wilcoxon test in Table 11, it is verified that the proposed algorithm is statistically meaningful. The reason is that all p values are less than 0.05 except PSOBFO in F1, F2, F3, and F4. According to the p values in Table 12, all values are less than 0.05 except PSOBFO in F11 problem. Hence, it can be approved that the results of the CBFO are statistically improved compared to the other methods. As can be seen from the p value in Table 13, the CBFO algorithm is significantly better than the PSOBFO, FPA, BA, and PSO for F14-F23.

The results demonstrate that the utilized chaotic mapping strategy and Gaussian mutation in the CBFO technique have improved the efficacy of the classical BFO, in a significant manner. On the one hand, applying the chaotic mapping strategy to the bacterial population initialization process can speed up the initial exploration of the algorithm. On the other hand, adding Gaussian mutation to the current best bacterial individual in the iterative process helps to jump out of the local optimum. In conclusion, the proposed CBFO can make a better balance between explorative and exploitative trends using the embedded strategies.

### 5.2. Results on the Parkinson's Disease

Many studies have demonstrated that the performance of BFO can be affected heavily by the chemotaxis step size* C*(*i*). Therefore, we have also investigated the effects of* C*(*i*) on the performance of the CBFO-FKNN.  Table 14 displays the detailed results of CBFO-FKNN model with different values of* C*(*i*) on the two datasets. In the table, the mean results and their standard deviations (in parentheses) are listed. As shown, the CBFO-FKNN model performed best with an average accuracy of 96.97%, an AUC of 0.9781, a sensitivity of 96.87%, and a specificity of 98.75% when* C*(*i*) = 0.1 on the Oxford dataset and an average accuracy of 83.68%, an AUC of 0.6513, a sensitivity of 96.92%, and a specificity of 33.33% when* C*(*i*) = 0.2 on the Istanbul dataset. Furthermore, the CBFO-FKNN approach also yielded the most reliable results with the minimum standard deviation when* C*(*i*) = 0.1 and* C*(*i*) = 0.2 on the Oxford dataset and Istanbul dataset, respectively. Therefore, values of 0.1 and 0.2 were selected as the parameter value of* C*(*i*) for CBFO-FKNN on the two datasets, respectively, in the subsequent experimental analysis.

The ACC, AUC, sensitivity, specificity, and optimal (*k*,* m*) pair values of each fold obtained via the CBFO-FKNN model with* C*(*i*) = 0.1 and* C*(*i*) = 0.2 on the Oxford dataset and Istanbul dataset are shown in Tables 15 and 16, respectively. As shown, each fold possessed a different parameter pair (*k*,* m*) since the parameters for each set of fold data were automatically determined via the CBFO method. With the optimal parameter pair, the FKNN yielded different optimal classification performance values in each fold. This was attributed to the adaptive tuning of the two parameters by the CBFO based on the specific distribution of each data set.

**Figure 5:**
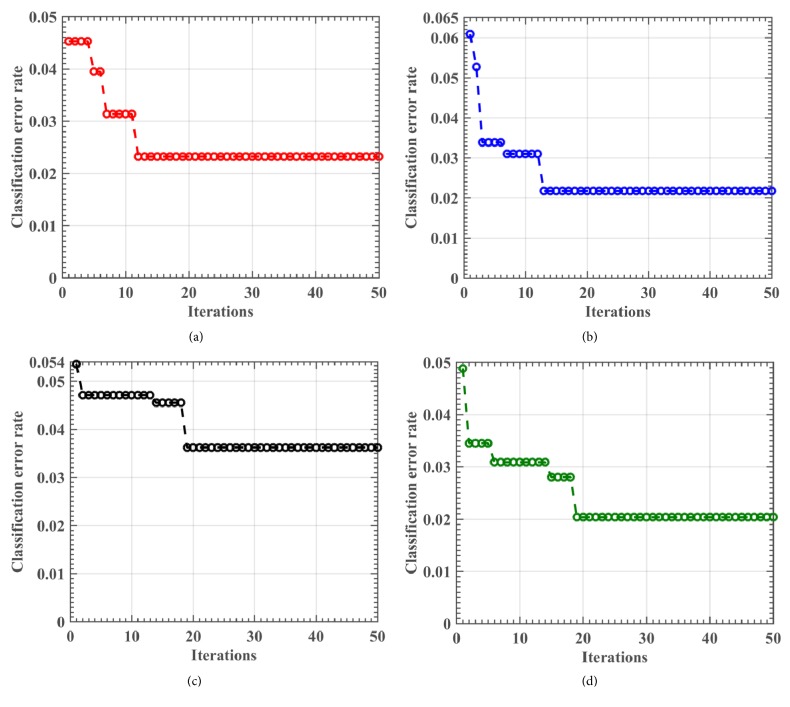
Learning curves of CBFO for fold 2 (a), fold 4 (b), fold 6 (c), and fold 8 (d) during the training stage.

In order to investigate the convergence behavior of the proposed CBFO-FKNN method, the classification error rate versus the number of iterations was recorded. For simplicity, herein we take the Oxford dataset for example. Figures 5(a)–5(d) display the learning curves of the CBFO-FKNN for folds 1, 3, 5, and 7 in the 10-fold CV, respectively. As shown, all four fitness curves of CBFO converged into a global optimum in fewer than 20 iterations. The fitness curves gradually improved from iterations 1 through 20 but exhibited no significant improvements after iteration 20. The fitness curves ceased after 50 iterations (the maximum number of iterations). The error rates of the fitness curves decreased rapidly at the beginning of the evolutionary process and continued to decrease slowly after a certain number of iterations. During the latter part of the evolutionary process, the fitness curves remained stable until the stopping criteria, the maximum number of iterations, were satisfied. Thus, the proposed CBFO-FKNN model efficiently converged toward the global optima.

**Figure 6:**
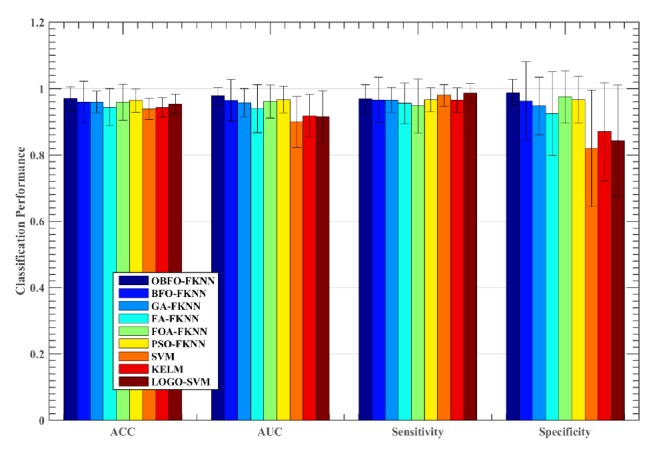
Comparison results obtained on the Oxford dataset by the nine methods.

**Figure 7:**
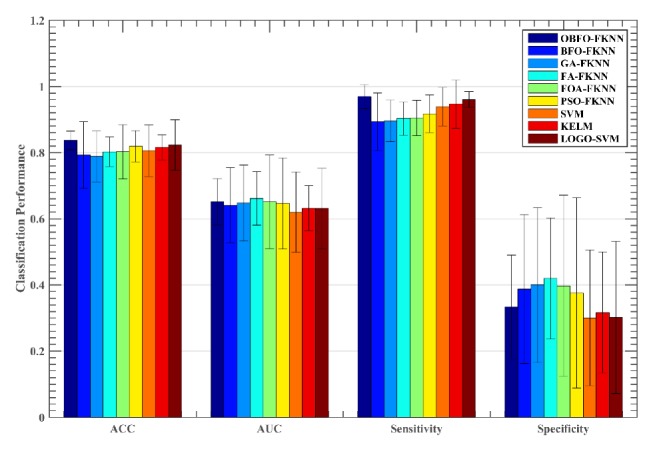
Comparison results obtained on the Istanbul dataset by the nine methods.

To validate the effectiveness of the proposed method, the CBFO-FKNN model was compared to five other meta-heuristic algorithms-based FKNN models as well as three other advanced machine learning approaches including SVM, KELM, and SVM with local learning-based feature selection (LOGO-SVM). As shown in Figure 6, the CBFO-FKNN method performed better than other competitors in terms of ACC, AUC, and sensitivity on the Oxford dataset. We can see that the CBFO-FKNN method yields the highest average ACC value of 96.97%, followed by PSO-FKNN, LOGO-SVM, KELM, SVM, FOA-FKNN, FA-FKNN, and BFO-FKNN. GA-FKNN has got the worst result among the all methods. On the AUC metric, OBF-FKNN obtained similar results with FA-FKNN, followed by FOA-FKNN, GA-FKNN, PSO-FKNN, BFO-FKNN, KELM, and LOGO-SVM, and SVM has got the worst result. On the sensitivity metric, CBFO-FKNN has achieved obvious advantages, LOGO-FKNN ranked second, followed by KELM, SVM, PSO-FKNN, FOA-FKNN, FA-FKNN, and GA-FKNN. BFO-FKNN has got the worst performance. On the specificity metric, FA-FKNN achieved the maximum results, GA-FKNN and FOA-FKNN have achieved similar results, which ranked second, followed by BFO-FKNN, PSO-FKNN, CBFO-FKNN, and SVM. KELM and LOGO-SVM have obtained similar results, both of which got the worst performance. Regarding the Istanbul dataset, CBFO-FKNN produced the highest result with the ACC of 83.68%, while the LOGO-SVM and PSO-FKNN method yields the second best average ACC value as shown in Figure 7, followed by KELM, SVM, FOA-FKNN, FA-FKNN, BFO-FKNN, and GA-FKNN. From Figures 6 and 7, we can also find that the CBFO-FKNN can yield a smaller or comparative standard deviation than the other counterparts in terms of the four performance metrics on the both datasets. Additionally, we can find that the SVM with local learning-based feature selection can improve the performance of the two datasets. It indicates that there are some irrelevant features or redundant features in these two datasets. It should be noted that the LOGO method was used for feature selection, all the features were ranked by the LOGO, then all the feature subsets were evaluated incrementally, and finally the feature subset achieved the best accuracy was chosen as the one in the experiment.

**Figure 8:**
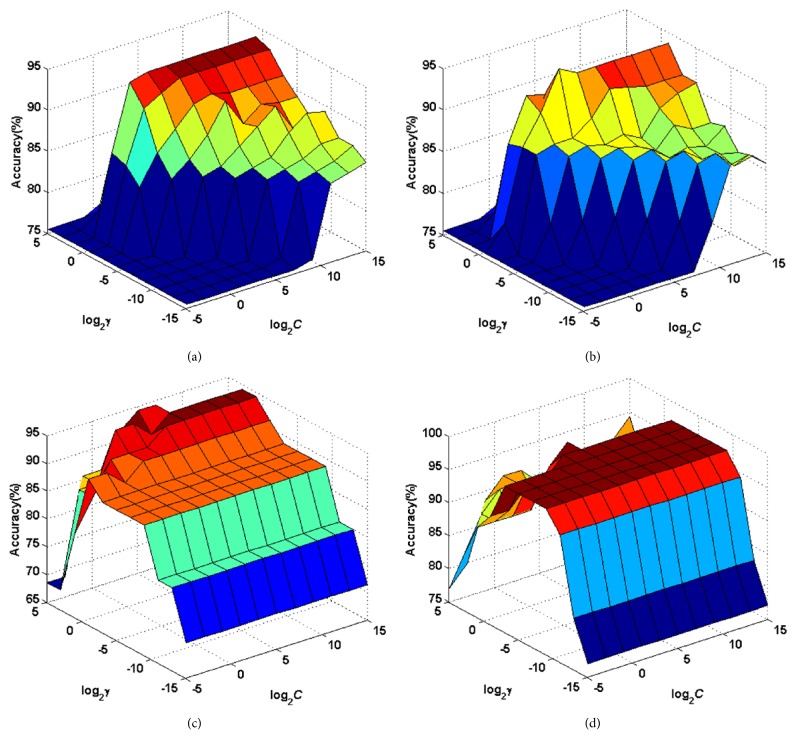
Training accuracy surfaces of SVM and KELM via the grid search method on the Oxford dataset. (a) Fold 2 for SVM. (b) Fold 4 for SVM on the data. (c) Fold 6 for KELM on the data. (d) Fold 8 for SVM on the data.

According to the results, the superior performance of the proposed CBFO-FKNN indicates that the proposed method was the most robust tool for detection of PD among the nine methods. The main reason may lie in that the OBL mechanism greatly improves the diversity of the population and increases the probability of BFO escaping from the local optimum. Thus, it gets more chances to find the optimal neighborhood size and fuzzy strength values by the CBFO, which aided the FKNN classifier in more efficiently achieving the maximum classification performance.  Figure 8 displays the surface of training classification accuracies achieved by the SVM and KELM methods for several folds of the training data via the grid search strategy on the Oxford dataset. Through the experimental process, we can find the original BFO is more prone to overfitting; this paper introduces chaotic initialization, enriches the diversity of the initial population, and improves the convergence speed of the population as well; in addition, this paper also introduced Gaussian mutation strategy for enhancing the ability of the algorithm to jump out of local optimum, so as to alleviate the overfitting problem of FKNN in the process of classification.

We have also investigated whether the diagnosis was affected by age and gender. Herein, we have taken the Oxford dataset for example. The dataset was divided by the age (old or young) and gender (male or female), respectively. Regarding the age, we have chosen the mean age of 65.8 years as the dividing point. The samples in the old group are more than 65.8, and the samples in the young group are less than 65.8. Therefore, we can obtain four groups of data including male group, female group, old group, and young group. The classification results of the four groups in terms of confusion matrix are displayed in Table 17. As shown, we can find that either in the male group or in the female group 3 PD samples were wrongly classified as healthy ones, and 2 healthy samples were misjudged as PD ones. It indicates that the gender has little impact on the diagnostic results. In the old group, we can find that 4 PD samples were wrongly identified as healthy ones. However, none of the samples were misjudged in the young group. It suggests that the speech samples in the old group are much easier to be wrongly predicted than those in the young group.

To further investigate the impact of gender and age on the diagnosis results. We have further divided the samples into male group and female group on the premise of young and old age and old group and young group on the premise of male and female, respectively. So we can obtain 8 groups as shown in Table 18, and the detailed classification results are displayed in terms of confusion matrix. As shown, we can find that the probability of the sample being misclassified is closer in the old group and young group on the premise of male and female. It can be also observed that there was no sample being wrongly predicted in male and female groups on the premise of young persons, while there was one sample being wrongly predicted in male and female groups on the premise of old persons, respectively. We can arrive at the conclusion that the presbyphonic may play a confounding role in the female and male dysphonic set, and the results of diagnosis were less affected by gender.

The classification accuracies of other methods applied to the diagnosis of PD are presented for comparison in Table 19. As shown, the proposed CBFO-FKNN method achieved relatively high classification accuracy and, therefore, it could be used as an effective diagnostic tool.

## 6. Conclusions and Future Work

In this study, we have proposed a novel evolutionary instance-based approach based on a chaotic BFO and applied it to differentiating the PD from the healthy people. In the proposed methodology, the chaos theory enhanced BFO strategy was used to automatically determine the two key parameters, thereby utilizing the FKNN to its fullest potential. The results suggested that the proposed CBFO-FKNN approach outperformed five other FKNN models based on nature-inspired methods and three commonly used advanced machine learning methods including SVM, LOGO-SVM, and KELM, in terms of various performance metrics. In addition, the simulation results indicated that the proposed CBFO-FKNN could be used as an efficient computer-aided diagnostic tool for clinical decision-making. Through the experimental analysis, we can arrive at the conclusion that the presbyphonic may play a confounding role in the female and male dysphonic set, and the results of diagnosis were less affected by gender. Additionally, the speech samples in the old group are much easier to be wrongly predicted than those in the young group.

In future studies, the proposed method will be implemented in a distributed environment in order to further boost its PD diagnostic efficacy. Additionally, implementing the feature selection using CBFO strategy to further boost the performance of the proposed method is another future work. Finally, due to the small vocal datasets of PD, we will generalize the proposed method to much larger datasets in the future.

## Figures and Tables

**Algorithm 1:**
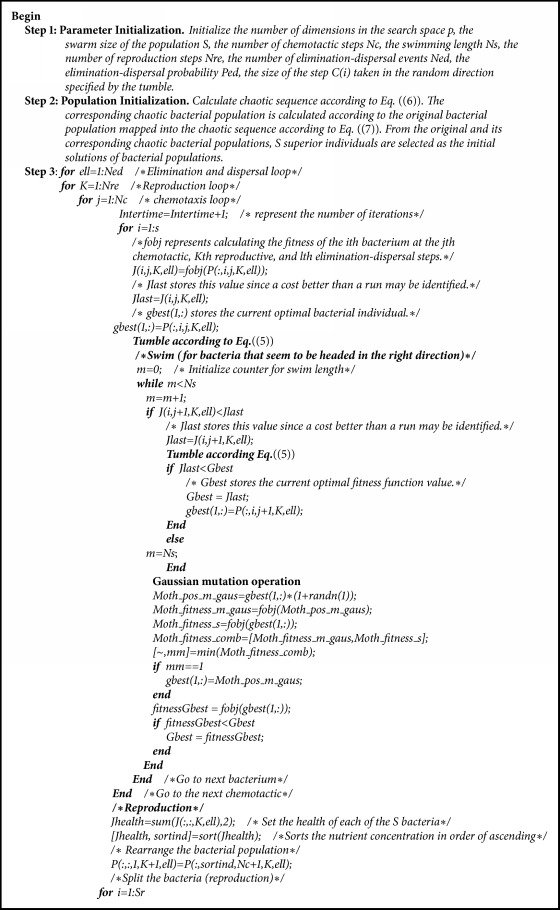
The steps of CBFO.

**Table 1:** Description of the Oxford PD data set.

Label	Feature
S1	MDVP:Fo(Hz)
S2	MDVP:Fhi(Hz)
S3	MDVP:Flo(Hz)
S4	MDVP:Jitter(%)
S5	MDVP:Jitter(Abs)
S6	MDVP:RAP
S7	MDVP:PPQ
S8	Jitter:DDP
S9	MDVP:Shimmer
S10	MDVP:Shimmer(dB)
S11	Shimmer:APQ3
S12	Shimmer:APQ5
S13	MDVP:APQ
S14	Shimmer:DDA
S15	NHR
S16	HNR
S17	RPDE
S18	D2
S19	DFA
S20	Spread1
S21	Spread2
S22	PPE

**Table 2:** Description of the Istanbul PD data set.

Label	Feature
S1	Jitter(local)
S2	Jitter(local, absolute)
S3	Jitter(rap)
S4	Jitter(ppq5)
S5	Jitter(ddp)
S6	Number of pulses
S7	Number of periods
S8	Mean period
S9	Standard dev. of period
S10	Shimmer(local)
S11	Shimmer(local, dB)
S12	Shimmer(apq3)
S13	Shimmer(apq5)
S14	Shimmer(apq11)
S15	Shimmer(dda)
S16	Fraction of locally unvoiced frames
S17	Number of voice breaks
S18	Degree of voice breaks
S19	Median pitch
S20	Mean pitch
S21	Standard deviation
S22	Minimum pitch
S23	Maximum pitch
S24	Autocorrelation
S25	Noise-to-Harmonic
S26	Harmonic-to-Noise

**Table 3:** Parameter setting of other optimizers involved in training FKNN.

**Parameters**	**GA**	**PSO**	**FA**	**FOA**
Population size	8	8	8	8
Max iteration	250	250	250	250
Search space	[2^−8^, 2^8^]	[2^−8^, 2^8^]	[2^−8^, 2^8^]	[2^−8^, 2^8^]
Crossover rate	0.8	-	-	-
Mutation rate	0.05	-	-	-
Acceleration constants	-	2	-	-
Inertia weight	-	1	-	-
Differential weight			-	-
Alpha	-	-	0.5	-
Beta	-	-	0.2	-
Gamma	-	-	1	-
*ax*	-	-	-	20
*bx*	-	-	-	10
*ay*	-	-	-	20
*by*	-	-	-	10

**Table 4:** Unimodal benchmark functions.

Function	Dim	Range	*f*_min_
$f_{1}\left(x\right)=\overset{n}{\underset{i=1}{\sum }}{x_{i}}^{2}$	30	[-100, 100]	0
$f_{2}\left(x\right)=\overset{n}{\underset{i=1}{\sum }}\left|x_{i}\right|+\overset{n}{\underset{i=1}{\prod }}\left|x_{i}\right|$	30	[-10, 10]	0
$f_{3}\left(x\right)=\overset{n}{\underset{i=1}{\sum }}{\left(\sum_{j-1}^{i}x_{j}\right)}^{2}$	30	[-100, 100]	0
$f_{4}\left(x\right)=\underset{i}{\max}\left\{\left|x_{i}\right|,\;\;1\leq i\leq n\right\}$	30	[-100, 100]	0
$f_{5}\left(x\right)=\overset{n-1}{\underset{i=1}{\sum }}[100(x_{i+1}-{x_{i}}^{2}{)}^{2}+\left(x_{i}-1{)}^{2}\right]$	30	[-30, 30]	0
$f_{6}\left(x\right)=\overset{n}{\underset{i=1}{\sum }}([x_{i}+0.5]{)}^{2}$	30	[-100, 100]	0
$f_{7}\left(x\right)=\overset{n}{\underset{i=1}{\sum }}i{x_{i}}^{4}+random[0,1)$	30	[-1.28, 1.28]	0

**Table 5:** Multimodal benchmark functions.

Function	Dim	Range	*f*_min_
$f_{8}\left(x\right)=\overset{n}{\underset{i=1}{\sum }}-x_{i}\sin\left(\sqrt{\left|x_{i}\right|}\right)$	30	[-500,500]	-418.9829*∗*5
$f_{9}\left(x\right)=\overset{n}{\underset{i=1}{\sum }}\left[{x_{i}}^{2}-10\cos\left(2\pi x_{i}\right)+10\right]$	30	[-5.12,5.12]	0
$f_{10}\left(x\right)=-20\exp\left(-0.2\sqrt{\frac{1}{n}\overset{n}{\underset{i=1}{\sum }}{x_{i}}^{2}}\right)-exp\left(\frac{1}{n}\overset{n}{\underset{i=1}{\sum }}\cos\left(2\pi x_{i}\right)\right)+20+e$	30	[-32,32]	0
$f_{11}\left(x\right)=\frac{1}{4000}\overset{n}{\underset{i=1}{\sum }}{x_{i}}^{2}-\overset{n}{\underset{i=1}{\prod }}cos\left(\frac{x_{i}}{\sqrt{i}}\right)+1$	30	[-600,600]	0
$f_{12}\left(x\right)=\frac{\pi }{n}\left\{10\sin\left(\pi y_{1}\right)+\overset{n-1}{\underset{i=1}{\sum }}{\left(y_{i}-1\right)}^{2}\left[1+10\;sin^{2}\left(\pi y_{i+1}\right)\right]+(y_{n}-1{)}^{2}\right\}$	30	[-50,50]	0
$+\overset{n}{\underset{i=1}{\sum }}u\left(x_{i},10,100,4\right)$			
$y_{i}=1+\frac{x_{i}+1}{4}u\left(x_{i},a,k,m\right)\left\{\begin{array}{cc} k\left(x_{i}-a\right) & x_{i}>a\\ 0 & -a<x_{i}<a\\ k\left(x_{i}-a\right) & x_{i}<-a \end{array}\right.$			
$f_{13}\left(x\right)=0.1\left\{sin^{2}\left(3\pi x_{1}\right)+\overset{n}{\underset{i=1}{\sum }}{\left(x_{i}-1]\right)}^{2}\left[1+sin^{2}\left(3\pi x_{i}+1\right)\right]+{\left(x_{n}-1\right)}^{2}\left[1+sin^{2}\left(2\pi x_{n}\right)\right]\right\}$	30	[-50,50]	0
$+\overset{n}{\underset{i=1}{\sum }}u(x_{i},5,100,4)$			

**Table 6:** Fixed-dimension multimodal benchmark functions.

Function	Dim	Range	*f*_min_
$f_{14}\left(x\right)={\left(\frac{1}{500}+\sum_{j=1}^{25}\frac{1}{j+\sum_{i=1}^{2}{\left(x_{i}-a_{ij}\right)}^{6}}\right)}^{-1}$	2	[-65,65]	1
$f_{15}\left(x\right)=\overset{11}{\underset{i=1}{\sum }}{\left[a_{i}-\frac{x_{1}\left(b_{i}^{2}+b_{i}x_{2}\right)}{b_{i}^{2}+b_{i}x_{3}+x_{4}}\right]}^{2}$	4	[-5, 5]	0.00030
$f_{16}\left(x\right)=4x_{1}^{2}-2.1x_{1}^{4}+\frac{1}{3}x_{1}^{6}+x_{1}x_{2}-4x_{2}^{2}+4x_{2}^{4}$	2	[-5,5]	-1.0316
$f_{17}\left(x\right)={\left(x_{2}-\frac{5.1}{4\pi^{2}}x_{1}^{2}+\frac{5}{\pi }x_{1}-6\right)}^{2}+10\left(1-\frac{1}{8\pi }\right)\cos x_{1}+10$	2	[-5,5]	0.398
*f*_18_(*x*) = [1 + (*x*_1_ + *x*_2_ + 1)^2^(19 − 14*x*_1_ + 3*x*_1_^2^ − 14*x*_2_ + 6*x*_1_*x*_2_ + 3*x*_2_^2^)]	2	[-2,2]	3
×[30 + (2*x*_1_ − 3*x*_2_)^2^*times*(18 − 32*x*_1_ + 12*x*_1_^2^ + 48*x*_2_ − 36*x*_1_*x*_2_ + 27*x*_2_^2^)]			
$f_{19}\left(x\right)=-\overset{4}{\underset{i=1}{\sum }}c_{i}\exp\left(-\overset{3}{\underset{j=1}{\sum }}a_{ij}{\left(x_{j}-p_{ij}\right)}^{2}\right)$	3	[1,3]	-3.86
$f_{20}\left(x\right)=-\overset{4}{\underset{i=1}{\sum }}c_{i}\exp\left(-\overset{6}{\underset{j=1}{\sum }}a_{ij}{\left(x_{j}-p_{ij}\right)}^{2}\right)$	6	[0,1]	-3.32
$f_{21}\left(x\right)=-\overset{5}{\underset{i=1}{\sum }}{\left[\left(X-a_{i}\right){\left(X-a_{i}\right)}^{T}+c_{i}\right]}^{-1}$	4	[0,10]	-10.1532
$f_{22}\left(x\right)=-\overset{7}{\underset{i=1}{\sum }}{\left[\left(X-a_{i}\right){\left(X-a_{i}\right)}^{T}+c_{i}\right]}^{-1}$	4	[0,10]	-10.4028
$f_{23}\left(x\right)=-\overset{10}{\underset{i=1}{\sum }}{\left[\left(X-a_{i}\right){\left(X-a_{i}\right)}^{T}+c_{i}\right]}^{-1}$	4	[0,10]	-10.5363

**Table 7:** Parameters setting for the involved algorithms.

Method	Population size	Maximum generation	Other parameters
BFO	50	500	Δ ∈ [-1, 1]
BA	50	500	*Q Frequency∈*[0 2]; *A Loudness*: 0.5; *r Pulse rate*: 0.5
DA	50	500	*w* ∈ [0.9 0.2]; *s* = 0.1; a = 0.1; *c* = 0.7; *f* = 1; *e* = 1
FA	50	500	*β*_0_=1; *α* ∈ [0 1]; *γ*=1
FPA	50	500	*switch probability p*=0.8; *λ*=1.5
PSO	50	500	*inertial weight=*1; *c*_1_=2; *c*_2_=2
PSOBFO	50	500	*inertial weight=*1; *c*_1_=1.2; *c*_2_=0.5; Δ ∈ [-1, 1]

**Table 8:** Results of unimodal benchmark functions (F1-F7).

F		CBFO	PSOBFO	BFO	FA	FPA	BA	DA	PSO
F1	Avg	0	0	8.73E-03	9.84E-03	1.45E+03	1.70E+01	2.15E+03	1.45E+02
	Stdv	0	0	3.85E-03	3.20E-03	4.07E+02	2.09E+00	1.13E+03	1.56E+01
	Rank	1	1	3	4	7	5	8	6

F2	Avg	0	0	3.55E-01	3.88E-01	4.59E+01	3.32E+01	1.53E+01	1.65E+02
	Stdv	0	0	7.44E-02	8.27E-02	1.49E+01	3.35E+01	6.54E+00	2.87E+02
	Rank	1	1	3	4	7	6	5	8

F3	Avg	0	0	4.96E-12	2.59E+03	1.99E+03	1.15E+02	1.46E+04	5.96E+02
	Stdv	0	0	8.97E-12	8.38E+02	4.84E+02	3.68E+01	8.91E+03	1.57E+02
	Rank	1	1	3	7	6	4	8	5

F4	Avg	0	0	3.24E-02	8.43E-02	2.58E+01	3.78E+00	2.95E+01	4.94E+00
	Stdv	0	0	5.99E-03	1.60E-02	3.96E+00	3.02E+00	8.22E+00	4.34E-01
	Rank	1	1	3	4	7	5	8	6

F5	Avg	2.90E+01	0	6.55E+04	2.33E+02	2.57E+05	4.48E+03	4.96E+05	1.77E+05
	Stdv	2.62E-02	0	NA	4.30E+02	1.88E+05	1.24E+03	6.46E+05	4.95E+04
	Rank	2	1	5	3	7	4	8	6

F6	Avg	1.34E-01	3.71E-01	2.11E+03	1.14E-02	1.53E+03	1.70E+01	2.06E+03	1.39E+02
	Stdv	1.76E-02	5.99E-02	1.15E+04	4.71E-03	4.23E+02	2.51E+00	1.52E+03	1.67E+01
	Rank	2	3	8	1	6	4	7	5

F7	Avg	3.62E-04	4.88E-03	3.77E-03	1.08E-02	4.60E-01	1.89E+01	6.92E-01	1.05E+02
	Stdv	3.21E-04	3.44E-03	3.33E-03	2.79E-03	1.42E-01	2.00E+01	3.79E-01	2.44E+01
	Rank	1	3	2	4	5	7	6	8

Sum of ranks		9	11	27	27	45	35	50	44
Average rank		1.2857	1.5714	3.8571	3.8571	6.4286	5	7.1429	6.2857
Overall rank		1	2	3	3	7	5	8	6

**Table 9:** Results of multimodal benchmark functions (F8-F13).

F		CBFO	PSOBFO	BFO	FA	FPA	BA	DA	PSO
F8	Avg	-3.47E+04	-2.55E+03	-2.47E+03	-6.55E+03	-7.58E+03	-7.45E+03	-5.44E+03	-7.05E+03
	Stdv	1.79E+04	5.80E+02	5.25E+02	6.70E+02	2.12E+02	6.56E+02	5.55E+02	5.98E+02
	Rank	1	7	8	5	2	3	6	4

F9	Avg	-2.89E+02	-2.90E+02	-2.88E+02	3.37E+01	1.44E+02	2.73E+02	1.71E+02	3.78E+02
	Stdv	2.98E-01	0	8.61E-01	1.13E+01	1.68E+01	3.08E+01	4.15E+01	2.46E+01
	Rank	2	1	3	4	5	7	6	8

F10	Avg	-9.66E+12	-1.07E+13	-9.08E+12	5.47E-02	1.31E+01	5.56E+00	1.02E+01	8.71E+00
	Stdv	3.21E+11	3.97E-03	7.34E+11	1.31E-02	1.59E+00	3.77E+00	2.15E+00	3.94E-01
	Rank	2	1	3	4	8	5	7	6

F11	Avg	0	0	4.99E-03	6.53E-03	1.49E+01	6.35E-01	1.65E+01	1.04E+00
	Stdv	0	0	3.18E-03	2.63E-03	3.38E+00	6.31E-02	8.41E+00	6.33E-03
	Rank	1	1	3	4	7	5	8	6

F12	Avg	1.34E-11	1.27E-08	3.04E-10	2.49E-04	1.16E+02	1.33E+01	7.90E+04	5.49E+00
	Stdv	3.46E-11	2.02E-08	5.97E-10	1.06E-04	4.75E+02	4.93E+00	4.26E+05	9.04E-01
	Rank	1	3	2	4	7	6	8	5

F13	Avg	4.20E-02	9.92E-02	9.92E-02	3.18E-03	6.18E+04	2.77E+00	4.46E+05	2.90E+01
	Stdv	4.64E-02	2.52E-08	4.17E-10	2.53E-03	9.34E+04	4.37E-01	7.19E+05	6.58E+00
	Rank	2	3	3	1	7	5	8	6

Sum of ranks		9	16	22	22	36	31	43	35
Average rank		1.5000	2.6667	3.6667	3.6667	6.0000	5.1667	7.1667	5.8333
Overall rank		1	2	3	3	7	5	8	6

**Table 10:** Results of fixed-dimension multimodal benchmark functions (F14-F23).

F		CBFO	PSOBFO	BFO	FA	FPA	BA	DA	PSO
F14	Avg	9.83E+00	3.11E+00	2.96E+00	1.82E+00	1.04E+00	4.53E+00	1.30E+00	4.41E+00
	Stdv	4.51E+00	1.71E+00	2.22E+00	8.42E-01	1.56E-01	3.91E+00	6.96E-01	3.20E+00
	Rank	8	5	4	3	1	7	2	6

F15	Avg	4.33E-04	9.49E-04	6.24E-04	2.85E-03	7.44E-04	8.29E-03	3.73E-03	1.41E-03
	Stdv	1.65E-04	3.00E-04	2.25E-04	4.71E-03	1.41E-04	1.35E-02	5.95E-03	4.04E-04
	Rank	1	4	2	6	3	8	7	5

F16	Avg	-1.03E+00	-1.03E+00	-1.03E+00	-1.03E+00	-1.03E+00	-1.03E+00	-1.03E+00	-1.03E+00
	Stdv	5.23E-06	1.60E-04	7.96E-06	3.36E-09	2.55E-08	8.94E-04	3.47E-06	2.49E-03
	Rank	1	1	1	1	1	1	1	1

F17	Avg	3.98E-01	3.98E-01	3.98E-01	3.98E-01	3.98E-01	3.98E-01	3.98E-01	3.99E-01
	Stdv	2.24E-06	4.80E-05	2.02E-06	1.76E-09	6.28E-09	5.45E-04	1.84E-07	1.65E-03
	Rank	1	1	1	1	1	1	1	1

F18	Avg	3.00E+00	3.01E+00	3.00E+00	3.00E+00	3.00E+00	3.10E+00	3.00E+00	3.24E+00
	Stdv	2.00E-04	6.53E-03	3.13E-04	2.59E-08	1.60E-06	8.65E-02	6.09E-07	3.61E-01
	Rank	1	6	1	1	1	6	1	8

F19	Avg	-3.86E+00	-3.86E+00	-3.86E+00	-3.86E+00	-3.86E+00	-3.83E+00	-3.86E+00	-3.84E+00
	Stdv	4.86E-04	4.56E-03	5.87E-04	1.03E-09	2.38E-06	2.49E-02	1.16E-03	2.10E-02
	Rank	1	1	1	1	1	8	1	7

F20	Avg	-3.29E+00	-3.24E+00	-3.27E+00	-3.28E+00	-3.31E+00	-2.89E+00	-3.25E+00	-2.71E+00
	Stdv	2.41E-02	2.38E-02	2.48E-02	6.10E-02	6.06E-03	1.31E-01	1.01E-01	3.54E-01
	Rank	2	6	4	3	1	7	5	8

F21	Avg	-6.03E+00	-1.01E+01	-9.80E+00	-7.92E+00	-1.01E+01	-4.64E+00	-6.61E+00	-3.67E+00
	Stdv	9.74E-01	4.27E-02	1.28E+00	3.47E+00	1.30E-01	2.43E+00	2.62E+00	1.31E+00
	Rank	6	1	3	4	1	7	5	8

F22	Avg	-6.45E+00	-1.01E+01	-1.02E+01	-9.89E+00	-1.02E+01	-5.03E+00	-7.35E+00	-4.33E+00
	Stdv	1.22E+00	9.60E-01	9.61E-01	1.94E+00	4.87E-01	2.93E+00	2.98E+00	1.67E+00
	Rank	6	3	1	4	1	7	5	8

F23	Avg	-6.91E+00	-9.73E+00	-9.98E+00	-1.05E+01	-1.02E+01	-5.36E+00	-6.35E+00	-4.42E+00
	Stdv	1.30E+00	1.82E+00	1.63E+00	1.07E-06	4.94E-01	2.90E+00	3.36E+00	1.33E+00
	Rank	5	4	3	1	2	7	6	8

Sum of ranks		32	32	21	25	13	59	34	60
Average rank		3.2	3.2	2.1	2.5	1.3	5.9	3.4	6
Overall rank		4	4	2	3	1	7	6	8

**Table 11:** The calculated p-values from the functions (F1-F7) for the CBFO versus other optimizers.

Problem	PSOBFO	BFO	FA	FPA	BA	DA	PSO
F1	**1**	1.73E-06	1.73E-06	1.73E-06	1.73E-06	1.73E-06	1.73E-06
F2	**1**	1.73E-06	1.73E-06	1.73E-06	1.73E-06	1.73E-06	1.73E-06
F3	**1**	1.73E-06	1.73E-06	1.73E-06	1.73E-06	1.73E-06	1.73E-06
F4	**1**	1.73E-06	1.73E-06	1.73E-06	1.73E-06	1.73E-06	1.73E-06
F5	1.73E-06	1.73E-06	6.04E-03	1.73E-06	1.73E-06	1.73E-06	1.73E-06
F6	1.73E-06	1.73E-06	1.73E-06	1.73E-06	1.73E-06	1.73E-06	1.73E-06
F7	1.92E-06	3.52E-06	1.73E-06	1.73E-06	1.73E-06	1.73E-06	1.73E-06

**Table 12:** The calculated p-values from the functions (F8-F13) for the CBFO versus other optimizers.

Problem	PSOBFO	BFO	FA	FPA	BA	DA	PSO
F8	1.73E-06	1.73E-06	1.73E-06	1.92E-06	1.73E-06	1.73E-06	1.92E-06
F9	1.73E-06	6.89E-05	1.73E-06	1.73E-06	1.73E-06	1.73E-06	1.73E-06
F10	1.73E-06	4.90E-04	1.73E-06	1.73E-06	1.73E-06	1.73E-06	1.73E-06
F11	**1**	1.73E-06	1.73E-06	1.73E-06	1.73E-06	1.73E-06	1.73E-06
F12	3.52E-06	5.79E-05	1.73E-06	1.73E-06	1.73E-06	1.73E-06	1.73E-06
F13	1.73E-06	1.92E-06	3.61E-03	1.73E-06	1.73E-06	1.73E-06	1.73E-06

**Table 13:** The calculated p-values from the functions (F14-F23) for the CBFO versus other optimizers.

Problem	PSOBFO	BFO	FA	FPA	BA	DA	PSO
F14	6.34E-06	6.98E-06	5.22E-06	3.18E-06	2.22E-04	1.73E-06	1.06E-04
F15	3.88E-06	8.31E-04	1.73E-06	1.24E-05	1.92E-06	4.29E-06	1.92E-06
F16	2.35E-06	**7.50E-01**	1.73E-06	1.73E-06	1.73E-06	1.97E-05	1.73E-06
F17	1.92E-06	**3.60E-01**	1.73E-06	1.73E-06	1.73E-06	2.60E-06	1.73E-06
F18	2.35E-06	**8.45E-01**	1.73E-06	1.73E-06	1.73E-06	1.73E-06	1.73E-06
F19	1.92E-06	**8.22E-02**	1.73E-06	1.73E-06	1.73E-06	8.19E-05	1.73E-06
F20	3.41E-05	8.94E-04	**6.44E-01**	2.60E-06	1.73E-06	**3.82E-01**	1.73E-06
F21	1.73E-06	2.13E-06	4.99E-03	1.73E-06	8.22E-03	**7.04E-01**	6.34E-06
F22	3.88E-06	2.60E-06	1.64E-05	1.73E-06	1.85E-02	**1.85E-01**	8.92E-05
F23	1.24E-05	2.60E-05	1.73E-06	1.73E-06	1.96E-02	**4.05E-01**	1.02E-05

**Table 14:** Detailed results of CBFO-FKNN with different values of *C*(*i*) on the two datasets.

*C*(*i*)	Oxford dataset				Istanbul dataset			
	ACC	AUC	Sen	Spec	ACC	AUC	Sen	Spec
0.05	0.9542	0.9417	0.9666	0.9167	0.8230	0.6180	0.9694	0.2667
	(0.0370)	(0.0774)	(0.0356)	(0.1620)	(0.0636)	(0.1150)	(0.0413)	(0.2108)
0.1	**0.9697**	**0.9781**	**0.9687**	**0.9875**	0.8054	0.5946	0.9559	0.2333
	**(0.0351)**	**(0.0253)**	**(0.0432)**	**(0.0395)**	(0.0414)	(0.0746)	(0.0297)	(0.1405)
0.15	0.9489	0.9479	0.9358	0.9600	0.8155	0.6074	0.9648	0.2500
	(0.0629)	(0.0609)	(0.1158)	(0.0843)	(0.0669)	(0.1204)	(0.0450)	(0.2257)
0.2	0.9589	0.9466	0.9600	0.9333	**0.8368**	**0.6512**	**0.9691**	**0.3333**
	(0.0469)	(0.0860)	(0.0555)	(0.1610)	**(0.0283)**	**(0.0698)**	**(0.0360)**	**(0.1571)**
0.25	0.9587	0.9459	0.9669	0.9250	0.8257	0.6385	0.9603	0.3167
	(0.0536)	(0.0901)	(0.0459)	(0.1687)	(0.0770)	(0.1560)	(0.0328)	(0.2987)
0.3	0.9639	0.9689	0.9670	0.9708	0.8090	0.6165	0.9478	0.2833
	(0.0352)	(0.0308)	(0.0454)	(0.0623)	(0.0439)	(0.1112)	(0.0534)	(0.2491)

**Table 15:** Detailed classification results of CBFO-FKNN on the Oxford dataset.

**Fold **	**CBFO-FKNN**					
**No.**	ACC	AUC	Sen	Spec	*k*	*m*
1	0.9474	0.9667	0.9333	1.0000	1	1.77
2	1.0000	1.0000	1.0000	1.0000	1	2.94
3	0.9500	0.9688	0.9375	1.0000	1	3.92
4	0.9500	0.9375	1.0000	0.8750	1	6.89
5	0.9500	0.9667	0.9333	1.0000	1	9.33
6	0.9000	0.9412	0.8824	1.0000	1	7.26
7	1.0000	1.0000	1.0000	1.0000	1	9.21
8	1.0000	1.0000	1.0000	1.0000	1	7.61
9	1.0000	1.0000	1.0000	1.0000	1	8.95
10	1.0000	1.0000	1.0000	1.0000	1	7.25
Mean	**0.9697**	**0.9781**	**0.9687**	**0.9875**	**1**	**6.51**

**Table 16:** Detailed classification results of CBFO-FKNN on the Istanbul dataset.

**Fold**	**CBFO-FKNN**					
**No.**	ACC	AUC	Sen	Spec	*k*	*m*
1	0.8571	0.7273	0.9545	0.5000	3	4.80
2	0.8276	0.5833	1.0000	0.1667	3	3.70
3	0.8276	0.7065	0.9130	0.5000	3	7.30
4	0.8276	0.5833	1.0000	0.1667	3	4.16
5	0.8966	0.7500	1.0000	0.5000	3	9.40
6	0.7931	0.6232	0.9130	0.3333	3	2.50
7	0.8621	0.7283	0.9565	0.5000	3	9.70
8	0.8276	0.5833	1.0000	0.1667	3	4.30
9	0.8276	0.5833	1.0000	0.1667	3	8.20
10	0.8214	0.6439	0.9545	0.3333	3	7.04
Mean	**0.8368**	**0.6513**	**0.9692**	**0.3333**	**3**	**6.11**

**Table 17:** The confusion matrix obtained by CBFO-FKNN via 10-fold CV for each group.

**Male**	Predicted PD	Predicted health
Actual PD	97	3
Actual health	2	16

**Female**	Predicted PD	Predicted health
Actual PD	44	3
Actual health	2	28

**Old**	Predicted PD	Predicted health
Actual PD	87	4
Actual health	0	18

**Young**	Predicted PD	Predicted health
Actual PD	56	0
Actual health	0	30

**Table 18:** The confusion matrix obtained by CBFO-FKNN for each group with precondition.

**Old**	**Male**	Predicted PD	Predicted health
	Actual PD	62	1
	Actual health	0	6
	**Female**	Predicted PD	Predicted health
	Actual PD	27	1
	Actual health	0	12

**Young**	**Male**	Predicted PD	Predicted health
	Actual PD	37	0
	Actual health	0	12
	**Female**	Predicted PD	Predicted health
	Actual PD	19	0
	Actual health	0	18

**Male**	**Old**	Predicted PD	Predicted health
	Actual PD	61	2
	Actual health	0	6
	**Young**	Predicted PD	Predicted health
	Actual PD	35	2
	Actual health	0	12

**Female**	**Old**	Predicted PD	Predicted health
	Actual PD	27	1
	Actual health	0	12
	**Young**	Predicted PD	Predicted health
	Actual PD	19	0
	Actual health	0	18

**Table 19:** Comparison of the classification accuracies of various methods.

**Study**	**Method**	**Accuracy (**%**)**
Little et al. (2009)	Pre-selection filter + Exhaustive search + SVM	91.4(bootstrap with 50 replicates)
Shahbaba et al. (2009)	Dirichlet process mixtures	87.7(5-fold CV)
Das (2010)	ANN	92. (hold-out)
Sakar et al. (2010)	Mutual information based feature selection + SVM	92.75(bootstrap with 50 replicates)
Psorakis et al. (2010)	Improved mRVMs	89.47(10-fold CV)
Guo et al. (2010)	GP-EM	93.1(10-fold CV)
Ozcift et al. (2011)	CFS-RF	87.1(10-fold CV)
Li et al. (2011)	Fuzzy-based non-linear transformation + SVM	93.47(hold-out)
Luukka (2011)	Fuzzy entropy measures + Similarity classifier	85.03(hold-out)
Spadoto et al. (2011)	Particle swarm optimization + OPF	73.53(hold-out)
	Harmony search + OPF	84.01(hold-out)
	Gravitational search algorithm + OPF	84.01(hold-out)
AStröm et al. (2011)	Parallel NN	91.20(hold-out)
Chen et al.(2013)	PCA-FKNN	96.07(10-fold CV)
Babu et al. (2013)	projection based learning for meta-cognitive radial basis function network (PBL-McRBFN)	99.35% (hold-out)
Hariharan et al. (2014)	integration of feature weighting method, feature selection method and classifiers	100%(10-fold CV)
Cai et al. (2017)	support vector machine (SVM) based on bacterial foraging optimization (BFO)	97.42%(10-fold CV)
This Study	CBFO-FKNN	97.89%(10-fold CV)
